# A Atenuação da Gordura Pericoronária na Tomografia Computadorizada Revela um Fator Culpado de Doença Arterial Coronariana Associada a Esteroides Anabólicos-Androgênicos

**DOI:** 10.36660/abc.20230843

**Published:** 2024-03-07

**Authors:** Jennifer Mancio

**Affiliations:** 1 Royal Brompton Hospital Londres Reino Unido Royal Brompton Hospital, Londres – Reino Unido; 2 Faculdade de Medicina do Porto Porto Portugal Faculdade de Medicina do Porto, Porto – Portugal

**Keywords:** Doença Arterial Coronariana, Tomografia Computadorizada por Raios X/métodos, Congeneres da Testoterona/toxicidade, Fatores de Risco, Placa Aterosclerótica/patologia

O uso ilícito de esteroides anabólico-androgênicos (EAA) é um problema crescente de saúde pública que afeta principalmente não-atletas, fisiculturistas e adultos jovens, incluindo mulheres.^1^ É difícil determinar quantas pessoas usam esteroides anabolizantes por razões não médicas. Em uma meta-análise populacional geral, 6,4% dos homens e 1,6% das mulheres afirmaram ter usado EAA em sua vida.^2^ No Brasil, onde a aparência estética, o culto ao corpo e a beleza desempenham um papel social e econômico especial, a prevalência do uso de EAA pode variar entre 2,1% e 31,6%, conforme a região.^3^ EAA são substâncias sintéticas relacionadas aos hormônios sexuais masculinos, principalmente à testosterona. Em doses normais e durante um curto período, os EAA podem melhorar a força muscular e aumentar a massa corporal magra, mas em doses mais elevadas (muitas vezes 100 vezes acima da dose clínica), os EAA estão associados a uma taxa de mortalidade 4,6 vezes superior em comparação com a população em geral.^4^ Vários casos de eventos cardiovasculares agudos, incluindo infarto do miocárdio e acidente vascular cerebral (alguns deles fatais), foram descritos na literatura.^5,6^ O principal mecanismo fisiopatológico por trás dessa associação em adultos jovens é a promoção de um perfil lipídico e metabólico adverso caracterizado por níveis elevados de colesterol LDL e diminuição do colesterol HDL e resistência à insulina, o que, em última análise, pode levar ao acúmulo de placa aterosclerótica.^5^ É importante ressaltar que a trombose sem aterosclerose subjacente ou vasoespasmo também é altamente possível em usuários de EAA, dado seu estado de hipercoagulabilidade e aumento da viscosidade sanguínea devido ao tromboxano A2 e à síntese de fibrinogênio, inibição da produção de prostaciclina e aumento da eritropoiese.^7^

Diagnosticar doença arterial coronariana (DAC) em usuários (e abusadores) de EAA pode ser um desafio dado seu risco muito baixo com base nos escores de risco clássicos. A angiocoronariografia por tomografia computadorizada (ACTC) é atualmente o método de imagem de primeira linha para investigação de pacientes com suspeita de DAC.^8^ Apesar de sua excelente resolução espacial, a avaliação visual da ACTC apresenta limitações na detecção de doença da parede arterial coronariana em estágios iniciais, quando as placas coronárias ainda não são visíveis ao olho humano. Pesquisadores da Universidade de Oxford mostraram que mais da metade dos eventos acontecem em pacientes com placas não obstrutivas.^9,10^ Nos últimos 5 anos, desde a publicação do estudo histórico CRISP-CT, Oikonomou et al.,^11^ demonstraram de forma detalhada que a inflamação coronária – a causa da formação e instabilidade da DAC – pode estar presente apesar da ausência de placas visíveis; eles descobriram que os sinais liberados por vasos inflamados no tecido adiposo perivascular circundante, bloqueia a diferenciação de pré-adipócitos perivasculares em adipócitos maduros e carregados de lipídios.^11^ Assim, pacientes com DAC acumulam tecido adiposo perivascular com adipocitos de menor tamanho e menor conteúdo lipídico próximo à parede vascular e, consequentemente, menores valores negativos de atenuação de gordura em comparação com vasos não doentes. Essas alterações na composição da gordura perivascular podem ser detetadas tridimensionalmente pelo Índice de Atenuação de Gordura (FAI) – um biomarcador derivado de ACTC desenvolvido para detectar DAC precoce.^11,12^ Resumidamente, FAI é o sinal médio de gordura perivascular ao redor do vaso que pode ser obtido em qualquer ACTC adquirida rotineiramente. Este biomarcador de inflamação coronária foi desenvolvido e validado em grandes coortes de pacientes europeus e americanos e posteriormente integrado (juntamente com os fatores de risco cardiovascular) num modelo prognóstico para obter o risco individual absoluto de eventos. O cálculo do FAI implica o contorno da parede dos vasos nas artérias coronárias contrastadas, o que é uma tarefa demorada e dependente do operador. Atualmente, uma ferramenta de IA está disponível para segmentar automaticamente a gordura perivascular e fornecer pontuação de risco de cada indivíduo para uso na prática clínica.

Nesta edição, De Souza et al.,^13^ compararam o FAI pericoronário derivado de ACTC e os níveis periféricos de Interleucina (IL) (IL-1, IL-6, IL-10) e TNF-alfa entre 20 usuários de EAA treinados em força, 20 não usuários de EAA e 10 controles sedentários. Os autores mostraram que os usuários de EAA apresentam maior FAI ao redor da artéria descendente anterior e coronariana direita em comparação com os não usuários de EAA ou controles, apesar de sua menor gordura corporal total. Notavelmente, os níveis circulantes de IL-1, IL-6 e IL-10 também são mais elevados em usuários de EAA. Esses achados demonstram que a inflamação sistêmica e coronariana (avaliada de forma não invasiva pelo FAI) está presente nos usuários de EAA, identificando assim a inflamação como um potencial alvo diagnóstico e terapêutico de comorbidades relacionadas à EAA. Nota-se o pequeno tamanho da coorte e a ausência de correlação de dados de resultados clínicos. No entanto, este estudo contribui para uma melhor compreensão dos fenómenos fisiopatológicos locais que ocorrem ao nível da parede coronária e alarga as aplicações do FAI a este grupo específico de jovens.

Como o abuso de EAA é uma preocupação crescente para os médicos, os cardiologistas devem considerá-lo como um possível diagnóstico diferencial no contexto de um paciente jovem que sofre um infarto do miocárdio com artérias coronárias normais (MINOCA). As imagens são mais do que podemos ver. Especificamente, em usuários de EAA, o FAI perivascular pode servir como uma ferramenta para detectar e monitorar a carga de inflamação da parede dos vasos coronários, independentemente da presença de placas coronárias visíveis. Futuros estudos maiores são necessários para corroborar os achados de De Souza et al.^13^ contra resultados clínicos após a retirada do EAA.
