# Supplementary material for: A Atenuação da Gordura Pericoronária na Tomografia Computadorizada Revela um Fator Culpado de Doença Arterial Coronariana Associada a Esteroides Anabólicos-Androgênicos
Source: Arq Bras Cardiol. 2024 Mar 7;120(11):e20230843. [Article in Portuguese] doi: 10.36660/abc.20230843 (PMC11098588; doi:10.36660/abc.20230843)
Supplement: Translation

# Translation: Pericoronary Fat Attenuation on Computed Tomography Unveils a Guilty Factor of Coronary Artery Disease Associated with Anabolic–Androgenic Steroids

**Authors:** Jennifer Mancio

**Affiliations:** 1 London United Kingdom Royal Brompton Hospital, London – United Kingdom; 2 Porto Portugal Faculdade de Medicina do Porto, Porto – Portugal

**Keywords:** Coronary Artery Disese, Tomography, X-Ray Computed/methods, Testosterone Congeners/toxicity, Risk Factors, Plaque Atherosclerotic/pathology

The illicit use of anabolic–androgenic steroids (AAS) is a growing public health problem that affects mainly nonathletes, bodybuilders, and young adults, including women.^1^ It is difficult to determine how many people use anabolic steroids for non-medical reasons. In a general population meta-analysis, 6.4% of males and 1.6% of females appealed to have used AAS in their lives.^2^ In Brazil, where aesthetic appearance, body cult, and beauty play a special social and economic role, the prevalence of AAS use can vary between 2.1% and 31.6%, according to the region.^3^ AAS are synthetic substances related to the male sex hormones, particularly testosterone. In normal doses and over a short time, AAS can improve muscle strength and increase lean body mass, but at higher doses (often 100 times above the clinical dose), AAS are associated with a 4.6-fold higher mortality rate compared to the general population.^4^ Several cases of acute cardiovascular events, including myocardial infarction and stroke (some of them fatal), have been described in the literature.^5,6^ The main pathophysiological mechanism behind this association in young adults is the promotion of an adverse lipidic and metabolic profile characterized by elevated LDL- and decreased HDL-cholesterol and insulin resistance, which, ultimately, can lead to atherosclerotic plaque build-up.^5^ Importantly, thrombosis without underlying atherosclerosis or vasospasm is also highly possible in AAS users, given its hypercoagulability state and increased blood viscosity because of thromboxane A2, and fibrinogen synthesis, inhibition of prostacyclin production, and increase erythropoiesis.^7^

Diagnosing coronary artery disease (CAD) in AAS users (and abusers) may be challenging given its very low risk based on the classical risk scores. Computed tomography coronary angiography (CTCA) is the current first-line imaging method for the investigation of patients with suspicion of CAD.^8^ Despite its excellent spatial resolution, visual CTCA assessment has limitations in detecting coronary artery wall disease at early stages, when coronary plaques are not yet visible to the human eye. Researchers from the University of Oxford showed that more than half of the events happen in patients with non-obstructive plaques.^9,10^ Over the past 5 years, since the publication of the landmark CRISP-CT study, Oikonomou et al.^11^ have comprehensively demonstrated that coronary inflammation – the driver of CAD formation and instability – may be present despite the absence of visible plaques; they discovered that signals released from inflamed vessels into the surrounding perivascular adipose tissue block the differentiation of perivascular pre-adipocytes into mature, lipid-laden adipocytes.^11^ Thus, patients with CAD accumulate perivascular adipose tissue with smaller adipocyte size and lower lipid content near the vascular wall and, consequently, less negative fat attenuation values compared with non-diseased vessels. These changes in the perivascular fat composition can be tri-dimensionally captured by the Fat Attenuation Index (FAI) – a CTCA-derived biomarker developed to detect early CAD.^11,12^ Briefly, FAI is the mean perivascular fat signal around the vessel that can be obtained in any routinely acquired CTCA. This biomarker of coronary inflammation was developed and validated in large cohorts of European and American patients and further integrated (along with the cardiovascular risk factors) into a prognostic model to obtain the absolute individual risk of events. FAI calculation implies vessel wall contouring in contrasted coronary arteries, which is a time-consuming and operator-dependent task. Currently, an AI-tool is available to automatically segment the perivascular fat and provide each subject risk score for use in clinical practice.

In this edition, De Souza et al.^13^ compared the CTCA-derived pericoronary FAI and the peripheral levels of Interleukin (IL) (IL-1, IL-6, IL-10) and TNF-alpha among 20 strength-trained AAS users, 20 AAS nonusers and 10 sedentary controls. The authors showed that AAS users exhibit higher FAI around the LAD and the RCA compared with AAS nonusers or controls, despite its lower total body fat. Remarkably, circulating levels of IL-1, IL-6, and IL-10 are also higher in AAS users. These findings demonstrate that systemic and coronary inflammation (assessed non-invasively by FAI) is present in AAS, thereby pining inflammation as a potential diagnostic and therapeutic target of AAS-related comorbidities. Note is made of the small cohort size and absence of clinical outcome data correlation. Nonetheless, this study is a contribution to a better understanding of the local pathophysiological phenomena happening at the coronary wall level, and it extends the applications of FAI to this specific group of young subjects.

As the abuse of AAS is a growing concern for clinicians, cardiologists must consider this as a possible differential diagnosis in the context of a young patient suffering a myocardial infarction with normal coronary arteries (MINOCA). Images are more than what we can see. Specifically, in AAS users, perivascular FAI can serve as a tool to detect and monitor the coronary vessel wall inflammation burden, regardless of the presence of visible coronary plaques. Future larger studies are needed to corroborate De Souza et al.^13^ findings against clinical outcomes following the withdrawal of AAS.
